# Eating disorders in premenstrual dysphoric disorder: a neuroendocrinological pathway to the pathogenesis and treatment of binge eating

**DOI:** 10.1186/s40337-018-0222-2

**Published:** 2018-10-25

**Authors:** Camilla Lindvall Dahlgren, Erik Qvigstad

**Affiliations:** 1 Regional Department for Eating Disorders, Division of Mental Health and Addiction, Oslo University Hospital, Ullevål Hospital, Oslo, Norway; 2 Department of Gynecology, Oslo University Hospital, Ullevål Hospital, Oslo, Norway; 3 Faculty of Medicine, University of Oslo, Oslo, Norway

**Keywords:** Binge eating, Treatment, Premenstrual dysphoric disorder, GnRH, Bilateral salpingo-oophorectomy

## Abstract

**Background:**

This case report details the presentation, treatment and post-operative outcome of an adult female with co-occurring binge eating disorder and premenstrual dysphoric disorder (PMDD).

**Case presentation:**

The patient, self-presenting for treatment, reported having struggled with severe, debilitating physical and psychological PMDD symptoms for nearly a decade. After having taken part in a number of unsuccessful first- and second line treatments in primary and secondary care, the patient was referred to tertiary care at the Department of Gynecology at Oslo University Hospital in Norway. Chemical menopause using a gonadotropin-releasing hormone (GnRH) agonist was induced, predicting the desired response (i.e. resolution of PMDD symptoms) to bilateral salpingo-oophorectomy (BSO). At three- and six months post BSO follow-up, the patient reported complete resolution of all reported PMDD symptoms including marked increase in appetite (i.e. hyperphagia), specific food cravings and auxiliary binge eating.

**Conclusions:**

To our knowledge, this is the first case documenting the recovery from an eating disorder following surgical ovarian suppression. Our findings lend supports to existing studies linking binge eating to hormonal changes in the mid-luteal phase of the menstrual cycle, and may help advance new treatment options for a selected, severely impaired group of females struggling with excessive appetite and binge eating due to fluctuations in ovarian activity.

## Background

Premenstrual dysphoric disorder (PMDD) is a DSM-5 [1] depressive disorder (for diagnostic criteria, see Table 1), and the most severe form for premenstrual distress [2] affecting 3–5% of pre-menopausal, menstruating females [3]. The disorder comprises a cluster of cyclically occurring affective, behavioral and somatic symptoms, with some the most essential features being marked depressed mood, anxiety, anger/irritability, decreased interest in daily activities, social withdrawal and appetite changes [4, 5]. The pathogenesis of PMDD is not fully understood, but it is likely to have multiple biological, psychological and sociocultural determinants [4]. In recent years, research has focused largely on the role of serotonin in the pathophysiology of PMDD, and the effect of neuromodulation on PMDD symptoms, in particular antidepressants in the selective serotonin reuptake inhibitor (SSRI) class [6]. Findings showing rapid symptom relief using SSRIs support the role of serotonin in the etiology of PMDD, and is currently the recommended first line treatment for the disorder [7]. However, not all patients respond to SSRI treatments [8], and for some, side effects may preclude ongoing therapy [9]. For a selected group of patients where SSRIs are not acceptable, suppression of cyclical ovarian activity using a gonadotropin-releasing hormone (GnRH) agonist is often the next step. In combination with a low-dose estrogen replacement, this approach is highly effective in reducing, often completely alleviating PMDD symptoms [6]. A small number of patients do, however, tolerate GnRH agonist treatment poorly, preventing long-term use. For these patients, bilateral salpingo-oophorectomy (BSO) (i.e. the removal of both ovaries and both fallopian tubes) to induce surgical menopause may be considered as a last resort measure [6]. The vast majority of studies documenting post-surgery outcome, including treatment satisfaction, support the use of BSO in the treatment of PMDD [10–12].

**Table 1:** Premenstrual Dysphoric Disorder (PMDD) diagnostic criteria according to the DSM-5

A.	In the majority of menstrual cycles, at least 5 symptoms must be present in the final week before the onset of menses, start to *improve* within a few days after the onset of menses, and become *minimal* or absent in the week postmenses.
B.	One (or more) of the following symptoms must be present:
	1. Marked affective lability (e.g., mood swings; feeling suddenly sad or tearful or increased sensitivity to rejection)*
	2. Marked irritability or anger or increased interpersonal conflicts*
	3. Markedly depressed mood, feelings of hopelessness or self-deprecating thoughts*
	4. Marked anxiety, tension and/or feelings of being keyed up or on edge*
C.	One (or more) of the following symptoms must additionally be present, to reach a total of *five* symptoms when combined with symptoms from Criterion B above.
	1. Decreased interest in usual activities (e.g. school, work, friends, hobbies)*
	2. Subjective difficulty in concentration*
	3. Lethargy, easy fatigability, or marked lack of energy*
	4. Marked change in appetite; overeating; or specific food cravings*
	5. Hypersomnia or insomnia*
	6. A sense of being overwhelmed or out of control*
	7. Physical symptoms such as breast tenderness or swelling, joint or muscle pain, a sensation of “bloating” or weight gain*
Note: The symptoms in Criteria A-C must have been met for most menstrual cycles that occurred in the preceding year.	
D.	The symptoms are associated with clinically significant distress or interference with work, school, usual social activities, or relationships with others (e.g., avoidance of social activities; decreased productivity and efficiency at work, school, or home)*
E.	The disturbance is not merely an exacerbation of the symptoms of another disorder, such as major depressive disorder, panic disorder, persistent depressive disorder (dysthymia), or a personality disorder (although it may co-occur with any of these disorders)*
F.	Criterion A should be confirmed by prospective daily ratings during at least two symptomatic cycles. (Note: The diagnosis may be made provisionally prior to this confirmation.)*
G.	The symptoms are not attributable to the physiological effects of a substance (e.g., a drug of abuse, a medication, other treatment) or another medical condition (e.g., hyperthyroidism)*

*Note*. In addition to listing DSM-5 PMDD criteria, Table 1 also gives a post hoc account of the patient’s symptomatology. Criteria met are marked with an asterisk (*), illustrating the fulfilment of *all* listed criteria, accompanied by the presence of *all* listed symptoms

The *severity* of symptoms is the key component of PMDD in that they cause clinically significant, often severely disabling, distress or interference in daily functioning. The most severe PMDD symptoms reported are often those related to mood (e.g. depression, anxiety, anger/irritability), but a number of somatic symptoms also contribute to luteal phase impairments. One of these is the marked change in appetite, overeating and/or specific food cravings (see Table 1, Criterion C4). Cyclic variations in food intake has been documented in several studies, with binge eating (i.e. eating large amounts of food within a limited period of time while experiencing feelings of loss of control) being more pronounced during the luteal phase [13, 14]. The link between excessive energy intake or hunger, i.e. hyperphagia [15], and binge eating in PMDD, however, remains elusive. It is not unlikely, though, that a proportion of those with PMDD who experience hyperphagia engage in binge eating behaviors, and that an additional few fulfill criteria for binge eating disorder (BED) and/or bulimia nervosa (BN). If binge eating can be fully explained by the presence of PMDD, i.e. that is state-dependent and only manifests when the disorder is active, one would expect individuals to “lose” their binge eating behavior when they no longer have PMDD. The current case report details the pre- and post-operative outcome of an adult female with co-occurring binge eating and PMDD who did, in fact, “lose” her eating disorder parallel to recovering from PMDD. To our knowledge, this is the first study to illustrate the immediate recovery (following DSM-5 criteria) from a long-term eating disorder following this surgical procedure.

## Case presentation

The patient was a 39-year old female, self-presenting for treatment for severe PMDD. Upon her own request, the patient had been referred from a secondary health care center specializing in OB-GYN (i.e. obstetrics and gynecology), to the Department of Gynecology at Oslo University Hospital (OUS). The aim of the consultation at OUS was to get a second opinion regarding having bilateral oophorectomy to treat her PMDD symptoms. The patient and her husband, voluntarily child-free, both took part in the medical consultation.

When presenting for treatment, the patient reported having struggled with PMDD for nearly a decade with the most debilitating symptoms being affective lability, irritability, anger and interpersonal conflicts, as well as depressed mood and anxiety. The symptoms had increased exponentially over the course of the previous 12–18 months, and were now severely impairing daily functioning. The patient had tried a number of conservative treatments such as oral contraceptives, individual therapy and couple’s counselling, none of which had had a positive impact with regards to PMDD symptoms. Five years prior to presenting for treatment at OUS, the patient had been prescribed intermittent luteal-onset dosing of Citalopram®, an anti-depressant drug in the SSRI class. Albeit “taking the edge off”, the patient reported that the antidepressants provided far from satisfactory effects, and that she had reached a point where the symptoms were so debilitating that she was no longer functioning emotionally, socially, nor professionally. As well as listing the DSM-5 PMDD criteria, Table 1 also gives a post hoc account of the patient’s symptomatology. Criteria met are marked with an asterisk (*), illustrating the fulfilment of *all* listed criteria, accompanied by the presence of *all* listed symptoms, an immense symptom burden by any standard.

### Symptom presentation - marked increase in appetite, food cravings and binge eating

In addition to the psychological symptoms for which the patient had sought treatment, she also reported having struggled with eating and weight problems since the onset of PMDD (no prior history of an eating disorder was reported). Over the years, these had worsened in parallel with the intensification of affective and behavioral problems. The patient described these problems as an overall, marked increase in appetite and specific food cravings which started intensifying at the time of ovulation, and then increased exponentially until the onset of the menstrual period. She described this pre-menstrual appetite as an “insatiable hunger” characterized by cravings for palatable foods high in carbohydrates (e.g. chocolate, bread, cakes/cookies and pastry) and fats (e.g. nuts and cheese). The patient further described the urge to eat as “impossible to resist”, and explained that once she had given in to the cravings, she was unable to control her food intake (i.e. stop eating). Episodes of uncontrollable food intake almost exclusively took place when the patient was home alone, where large amounts of highly palatable foods were consumed. Initially, food was consumed to satiate hunger and cravings. However, even after having consumed enough to cease hunger, the patient kept on eating, often until feeling uncomfortably full. Due to the fact that as little as one slice of bread, a hand full of nuts or one piece of chocolate could trigger over eating, the patient tried avoiding having “trigger foods” at home. However, this solution only partially helped the patient to resist over eating. Episodes of over eating had occurred almost once weekly over the past 5–6 years, and were associated with a strong sense of shame and disgust, and a feeling of having to compensate by intermittently restricting food intake. The patient, albeit with a normal weight of 65 kg (BMI = 23.9) when presenting for treatment, reported having gained 12 k since the onset of PMDD. Prior to the onset of PMDD, the patient’s weight had been stable at 53 kg (BMI = 19.5) for many years, and as such, she attributed her weight increase to the erratic eating behaviors. Overall, food, eating and weight were constantly on the patient’s mind as she tried to manage a perpetual cycle of restricting and over eating.

### Treatment – Bilateral Salpingo-Oophorectomy (BSO)

Bilateral salpingo-oophorectomy (BSO) in the treatment of PMDD is considered a last-resort measure [6], and includes the removal of both ovaries and both fallopian tubes to induce surgical menopause. In the current case, all conservative first- and second line treatments had failed, leading to the consideration of BSO. With the aim of predicting the patient’s response to surgery (and as a prerequisite before referring the patient for BSO), a 3-month trial of a GnRH agonist injection (Procren®, 3.75 mg monthly), combined with estrogen replacement (Progynova®, 2 mg daily) was instigated. Two weeks after the first injection, the patient started noticing a number of adverse effects such as dizziness, arthropathy in hands and feet and amnesia. The discomfort associated with the side effects was severe enough to warrant discontinuation of the GnRH agonist trial. Hence, only one of the three depot injections was given. On a positive note, the patient reported complete (albeit short-term) resolution of all the above mentioned PMDD symptoms, indicating a positive, predictive response to BSO. Two months after the discontinuation of the GnRH agonist trial, laparoscopic BSO was approved. To prevent the risk of reintroducing PMDD symptoms after the surgery (which may be a result of progesterone which is prescribed to prevent endometrial hyperplasia or malignancy) [9], the patient also had a (supracervical) hysterectomy.

### Retrospective eating disorder assessment

Albeit being a part of the collective PMDD symptoms, the patient was not treated primarily for problems related to eating. As such, assessment of eating disorder symptoms and attitudes was not performed pre-surgery. However, just short of 4 weeks post-surgery, the patient was retrospectively assessed (i.e. asked to report symptoms present over the past 3 months *prior to surgery*) using the semi-structured, diagnostic interview the Eating Disorder Assessment for DSM-5 (EDA-5) [16], and the diagnostic self-report questionnaire Eating Disorder Diagnostic Scale (EDDS) [17]. Both assessments yielded a subclinical binge eating disorder (BED), where the patient fulfilled all BED criteria, but reported less than one weekly objective binge eating episode the previous 3 months (the DSM-5 frequency criteria requires that binge eating occurs, on average, at least once a week the previous 3 months). Based on the pattern of food intake related cognitions and behaviors identified through the EDA-5 and the EDDS, the patient’s eating behaviors are hereafter referred to as *binge eating* rather than over eating.

### Post-operative outcome

Symptom burden was assessed four, eight and 12 weeks, as well as at 6 months-post-surgery. Already at the four-week post-op evaluation, the patient reported complete resolution of all previously reported PMDD symptoms. This was also the case eight and 12-weeks after surgery, as well as at the 6-month follow-up. The patient had stopped taking the SSRIs the day after the surgery, and had not felt the need to reintroduce them. Further, the estrogen replacement was well tolerated, with no reports of adverse effects. Paraphrasing the patient, she describing the time after the surgery as “a period of inner peace” that she had not experienced for years. As for the problems related to food, eating and weight, the patient reported a significant decrease in appetite, a complete absence of the aforementioned cravings, and zero binge eating episodes. She now described feeling full after eating “healthy sized” portions, and had successfully reintroduced some of the trigger foods that she had previously avoided (e.g. chocolates, bread, nuts and cheese). She also described a sense of relief as she was no longer constantly thinking of food, and reported eating when hungry, without experiencing loss of control. Follow-up EDA-5 and EDDS assessments were performed 12-weeks and 6 months post-operatively. None of these interviews yielded a full threshold, or a subclinical eating disorder diagnosis. An EDDS symptom composite score, indicating the patient’s overall level of eating pathology, was calculated pre-surgery, and at three and six months follow-up. The retrospective, pre-surgery assessment yielded a composite score of 25, whereas the follow-up composite scores had markedly decreased to seven at 3-month follow-up, and to five at 6-month follow-up. An overall symptom cut-off score of 16.5 has shown to accurately distinguish between patients with eating disorders and healthy controls following DSM-IV criteria [18].

## Discussion and conclusion

This case report details the presentation, treatment and follow-up assessment of an adult female suffering from long-term PMDD and subclinical BED. Following a number of unsuccessful first- and second line treatments in primary and secondary care, the patient was referred to tertiary care where she underwent BSO and hysterectomy. Three and six-months post-operatively, the patient reported complete resolution of all reported PMDD symptoms including hyperphagia and auxiliary binge eating. The patient’s overall level of eating disorder psychopathology had also reduced drastically as illustrated by the decrease in reported EDDS symptoms. Whereas existing studies report improvement in mood, general affect, well-being, life satisfaction, and overall quality of life [11, 12] following BSO for PMDD, there are no studies specifically addressing the effect of BSO on eating behaviors or eating disorders. To our knowledge, this is thus the first study to document the recovery from a long-standing eating disorder according to DSM-5 criteria, following BSO.

Traditionally, binge eating has been regarded as means of affect regulation [19] with biological and neurobiological factors shedding light on its etiology [20, 21], and sex hormone fluctuations explaining the luteal phase increase in binge eating frequency [13, 14]. The current study lends supports to existing studies linking binge eating to hormonal changes in the mid-luteal phase of the menstrual cycle [14, 22, 23]. In addition, our case report adds to the current scope of knowledge by illustrating how premenstrual hyperphagia and auxiliary binge eating plays a role in the development and maintenance of binge eating behaviors stretching beyond the peri-ovulatory phase of the menstrual cycle. As such, this study provides an add-on perspective to traditional etiological approaches, and by extension, may provide new insight when seeking to understand why traditional, psychological interventions fail for some with binge eating problems [24].

For women where cyclic binge eating and conjunct comorbidity severely impair daily functioning, and where all conventional PMDD and eating disorder treatment options have failed or proved insufficient, suppression of cyclical ovarian activity using a GnRH agonist (supplemented by low-dose estradiol) could prove as a useful treatment alternative. GnRH agonists are highly effective and for many women, long-term use combined with an appropriate, low dose estrogen replacement is both well tolerated [9] and well accepted [12]. In addition, GnRH agonist treatment is completely reversible with gonadal function reverting to normal within a few days following discontinuation of treatment [25] making it a far less invasive treatment option compared to BSO. BSO, on the other hand, is an irreversible surgical procedure precluding any chances of becoming naturally pregnant (in vitro fertilization is still possible). In conjunct with hysterectomy, which is often performed to facilitate hormone replacement therapy until natural menopausal age, a woman will not be able to bear children at all. As such, extreme caution should be exerted before considering this treatment option for BED. It should be considered only as a last resort, i.e. when all other evidence based eating disorder treatments have proved unsuccessful, and when a subsequent GnRH agonist administration has failed (such as presented in this case report). Further, it should be considered an alternative only for women who do not wish to conceive, or those who have already established their family.

Premenstrual dysphoric disorder and BED are both associated with substantial burden on physical and mental aspects of quality of life [26–28]. The two disorders are also, individually, associated with suicidality [29, 30], binge eating even below the threshold for BED, as well as impaired work productivity, increased works absenteeism [31] and increased healthcare costs [27]. When combined, we may expect an aggregated burden, stressing the importance of finding appropriate, long-lasting treatment options dealing with both conditions. The presented case shows the potential treatment benefits of inducing chemical menopause to help treat severe cases of PMDD and associated binge eating, and in extension, BSO as a last resort when all other treatment attempts have failed. It is important to note that since PMDD was first introduced in the DSM-5, some may not be aware of the link between PMDD and binge eating (behaviors), nor the full extent to which hormonal fluctuations impact the development and maintenance of such behaviors in females. With this case report, the authors wish to increase awareness of the potential co-occurrence of these two disorders and encourage future research studies to investigate the association between hyperphagia and binge eating behaviors, and the prevalence of co-morbid PMDD and binge eating. Future research should also aim to expand the concept of recovery by including additional psychological/cognitive indices, and extend the duration of post-surgery symptom assessment. Finally, our case report may help advance new treatment options for a selected, severely impaired group of females struggling with excessive appetite and binge eating due to fluctuations in ovarian activity.

## Strengths and limitations

This case report has a number of strengths but also some limitations to note. Firstly, to the authors’ knowledge, this is the first study describing co-occurring PMDD and binge eating, as well as the documentation of the recovery (based on the absence of eating disorder symptoms following DSM-5 criteria) from a long-standing eating disorder following BSO. As such, our results, albeit limited to merely one individual, bring novelty to the field by highlighting how the development and maintenance of, as well as the recovery from, an eating disorder may depend solely on the presence and resolution of a co-occurring mental health condition originating from sex hormone fluctuations. Secondly, the patient was administered repeated diagnostic assessments following the surgical procedure, none of which yielded a sub- or full threshold eating disorder diagnosis. This strengthens the hypothesized, causal relationship between PMDD and binge eating, although long-term follow-ups are required to confirm such an association. Limitations of the present case report include the retrospective nature of the eating disorder assessment, the lack of an updated EDDS DSM-5 cut-off score, and an objectively registered weight trajectory between PMDD onset and the surgical procedure. Also, as results are limited to one case only, further research is required to investigate the association between binge eating (disorder) and chemical ovarian suppression, and to better understand both treatment benefits and potential adverse effects of GnRH agonist treatment in this group of patients.

## Abbreviations

BED: Binge eating disorder
BMI: Body mass index
BSO: Bilateral Salpingo-Oophorectomy
DSM-5: The Diagnostic and Statistical Manual of Mental Disorders
EDA-5: the Eating Disorder Assessment for DSM-5
EDDS: the Eating Disorder Diagnostic Scale
EDE-Q: The Eating Disorder Examination Questionnaire
GnRH: Gonadotropin releasing hormone
OB-GYN: Obstetrics and gynecology
OUS: Oslo University Hospital
PMDD: Premenstrual dysphoric disorder
SSRI: Selective serotonin reuptake inhibitors
